# COVID-19 Pandemic–Related Exposures and Cognitive Function in Middle-Aged Women

**DOI:** 10.1001/jamanetworkopen.2025.5532

**Published:** 2025-04-17

**Authors:** Siwen Wang, Anthony Menor, Lori B. Chibnik, Jae H. Kang, Chirag M. Vyas, Deborah L. Blacker, Laura D. Kubzansky, Karestan C. Koenen, Andrea L. Roberts

**Affiliations:** 1 Department of Nutrition, Harvard T.H. Chan School of Public Health, Boston, Massachusetts; 2 Department of Environmental Health, Harvard T.H. Chan School of Public Health, Boston, Massachusetts; 3 Department of Epidemiology, Harvard T.H. Chan School of Public Health, Boston, Massachusetts; 4 Department of Psychiatry, Massachusetts General Hospital, Boston; 5 Department of Psychiatry, Harvard Medical School, Boston, Massachusetts; 6 Department of Neurology, Massachusetts General Hospital, Boston; 7 Department of Neurology, Harvard Medical School, Boston, Massachusetts; 8 Channing Division of Network Medicine, Department of Medicine, Brigham and Women’s Hospital and Harvard Medical School, Boston, Massachusetts; 9 Department of Social and Behavioral Science, Harvard T.H. Chan School of Public Health, Boston, Massachusetts

## Abstract

**Importance:**

The COVID-19 pandemic has been associated with risk factors for cognitive decline, such as bereavement and SARS-CoV-2 infection.

**Objective:**

To examine whether the COVID-19 pandemic and pandemic-related exposures are associated with cognitive function among middle-aged women.

**Design, Setting, and Participants:**

This cohort study analyzed data from the Nurses’ Health Study II, an ongoing study of registered nurses in the US. The present study focused on women aged 51 to 76 years who completed 2 to 8 objective cognitive assessments both prior to (October 1, 2014, to February 29, 2020) and during the COVID-19 pandemic (March 1, 2020, to September 30, 2022). Statistical analyses were performed from January 2023 to January 2025.

**Exposure:**

COVID-19 pandemic.

**Main Outcomes and Measures:**

Two standardized (ie, *z*-scored) composite cognitive scores (psychomotor speed and attention, learning and working memory) and a global score constituted the primary outcomes. Higher scores indicated better cognitive function. Cognitive function was assessed using the Cogstate Brief Battery, a computer-administered cognitive test battery. Participants completed cognitive assessments every 6 to 12 months.

**Results:**

A total of 5191 women (mean [SD] age at first cognitive assessment, 63.0 [4.8] years) completed both prepandemic and during-pandemic measures, contributing 23 678 cognitive assessments. After adjustment for age at cognitive assessment, educational level for both participants and their parents, cognitive test practice effects, and comorbidities (eg, diabetes, hypertension), no difference in cognitive function was observed between assessments taken during vs before the pandemic (psychomotor speed and attention: β = −0.01 SD [95% CI, −0.05 to 0.02 SD]; learning and working memory: β = 0.00 SD [95% CI, −0.03 to 0.03 SD]; global score: β = 0.00 SD [95% CI, −0.03 to 0.02 SD]). Among 4456 participants who responded to the COVID-19 substudy (ie, surveys about pandemic-related events), those with a history of SARS-CoV-2 infection (164 [3.7%]) or post–COVID-19 conditions (PCC; 62 [1.4%]), at a median (IQR) 20.0 (18.5-22.1) months after initial infection, had reduced cognitive function compared with women without infection or PCC; however, these differences did not reach statistical significance, and the wide CIs suggested considerable uncertainty.

**Conclusions and Relevance:**

This cohort study of middle-aged women found that the COVID-19 pandemic and pandemic-related events were not associated with cognitive decline up to 2.5 years after the onset of the pandemic. Future studies are needed to examine the long-term implications of SARS-CoV-2 infection and PCC for cognitive function.

## Key Points

**Question:**

Are the COVID-19 pandemic and pandemic-related events associated with worse cognitive function in women?

**Findings:**

In this cohort study of 5191 middle-aged women who completed 2 to 8 objective cognitive assessments both before and during the pandemic, there was no difference in cognitive function during the pandemic compared with the prepandemic period. History of SARS-CoV-2 infection or self-reported post–COVID-19 conditions showed no associations with cognitive scores, although these estimates had wide CIs.

**Meaning:**

The findings indicate that the COVID-19 pandemic and pandemic-related exposures were not associated with worse cognitive function in this nonclinical sample.

## Teaser

This cohort study examines whether COVID-19 pandemic–related exposures are associated with changes in cognitive function among women aged 51 to 76 years.

## Introduction

The COVID-19 pandemic is associated with increased prevalence of risk factors for cognitive decline, including social isolation^1^ and bereavement.^2,3^ COVID-19 illness has been linked to cognitive difficulties; brain fog lasting beyond the acute phase is commonly reported among individuals with post–COVID-19 condition (PCC).^4,5,6^

Many studies found that the COVID-19 pandemic was associated with worse cognitive function in older adults,^7,8,9,10,11,12,13,14^ with only a few studies indicating no association or an association with improved function.^15,16,17^ Media coverage of these studies emphasized possible population-wide, long-lasting decreases in cognitive function as an outcome.^18,19^ However, most studies with objective cognitive assessments before and after the pandemic were small (<400 participants)^7,8,9,11,12,13^ and ended follow-up in the first year of the pandemic, an early and severe phase that may not be comparable to later years.^14,16^ The only large, longitudinal study with prepandemic and during-pandemic cognitive assessments of the same individuals was conducted in the UK and ended follow-up in February 2022. This UK study of 3000 older adults included 1 prepandemic and 1 to 2 during-pandemic cognitive assessments per person and found the pandemic was associated with worsened executive function and working memory, although decline in executive function was observed only in the first year of the pandemic.^10^

Studies have generally reported an association of a history of SARS-CoV-2 infection with lower cognitive function.^7,8,9,10,20,21,22,23,24,25^ Few of these studies had preinfection and postinfection cognitive measurements in individuals,^9,10^ and most had fewer than 100 participants or were conducted in hospital settings^7,8,20,21,23^; thus, their relevance to the general population is unknown. It is critical to know whether the pandemic accelerated cognitive decline in nonclinical populations so that efforts can be made to understand and mitigate, where possible, the sources of this decline. It is also important to know whether the pandemic did not accelerate cognitive decline to ensure that individuals do not incorrectly attribute their cognitive decline to the pandemic and seek appropriate care.

Women aged 65 years or older bear a higher burden of cognitive disorders than do men of the same age range.^26,27,28,29^ Evidence also suggests that women have a 1.5- to 2-fold greater risk than men of developing PCC and its associated cognitive concern.^30,31^ Thus, identifying the implications of the pandemic for cognitive function in older women has high public health salience. In the present study, we leveraged data from a large population-based longitudinal cohort of women with objective cognitive assessments obtained before (October 1, 2014, to February 29, 2020) and during the COVID-19 pandemic (March 1, 2020, to September 30, 2022) to examine the associations of the pandemic and pandemic-related exposures with cognitive function. We hypothesized that (1) population-level cognitive function would be worse during vs before the pandemic; (2) individuals with prepandemic risk factors for cognitive decline (eg, diabetes, depression) would have worse cognitive function during vs before the pandemic compared with persons without these risk factors; and (3) specific pandemic-related exposures (eg, SARS-CoV-2 infection and PCC) would be associated with worse cognitive function.

## Methods

### Study Design and Participants

The cohort study protocol was approved by the Brigham and Women’s Hospital and the Harvard T.H. Chan School of Public Health Institutional Review Boards. Return of questionnaires from participants implied their informed consent. We followed the Strengthening the Reporting of Observational Studies in Epidemiology (STROBE) reporting guideline.

We used data from the Nurses’ Health Study II, an ongoing cohort study established in 1989, when 116 429 registered nurses aged 25 to 42 years living in the US were enrolled. Questionnaires are sent to participants biennially to collect lifestyle and health information. The response rate for each follow-up cycle exceeds 85%.

In 2014, 15 138 participants of the Nurses’ Health Study II joined a cognitive health substudy (eMethods in Supplement 1), with cognitive assessments at 6- or 12-month intervals for up to 24 months (hereafter wave 1).^32^ In 2018, 11 920 participants enrolled in wave 2, with follow-up every 12 months for up to 24 months (eFigure 1 in Supplement 1). After exclusion of cognitive assessments that failed integrity checks,^33^ 20 659 participants completed 53 532 cognitive assessments between October 1, 2014, and September 30, 2022 (eFigure 2 in Supplement 1). At cohort enrollment, participants in the cognitive substudy were more likely to be White individuals, had slightly higher neighborhood socioeconomic status, and were less likely to have chronic conditions (eTable 1 in Supplement 1).

**Figure 1.**
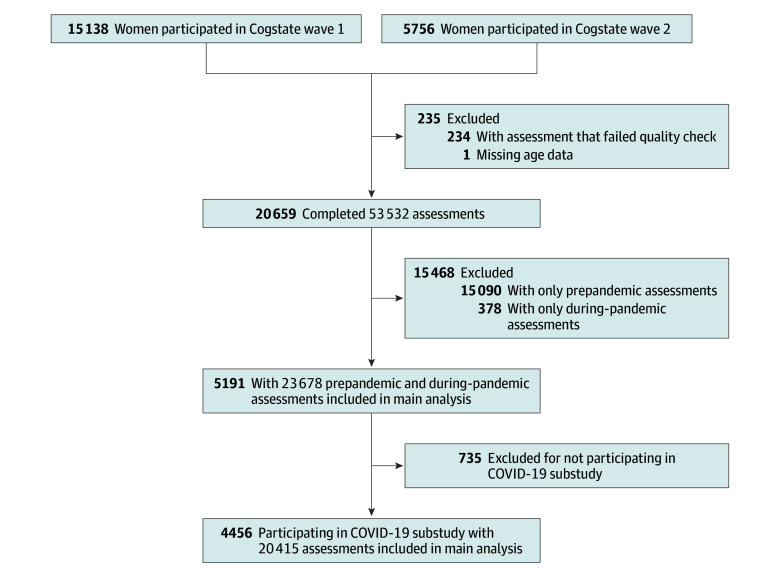
Flowchart of Study Design

The main analyses were restricted to 5191 women aged 51 to 76 years with both prepandemic and during-pandemic cognitive assessments (Figure 1; eTable 2 in Supplement 1). In April 2020, a COVID-19 substudy was launched to collect information on experiences during the pandemic,^34^ with the final questionnaire returned in November 2021. Participants in the COVID-19 substudy were included in the main analyses.

### Exposure Assessment

The primary exposure was the COVID-19 pandemic. We considered October 1, 2014, to February 29, 2020, as the prepandemic period and March 1, 2020, to September 30, 2022, as the during-pandemic period.

#### Risk Factors for Cognitive Decline

We considered hypertension, diabetes, stroke, depression, and cancer as possible cognitive risk factors. History of physician-diagnosed hypertension, diabetes, stroke, and cancer were self-reported biennially through 2017. Self-reported health outcomes have had high validity in cohorts of health professionals.^35,36^ Depression was derived from multiple indicators queried from 2010 to 2017, including self-reported physician-diagnosed depression, use of antidepressants, and depressive symptoms (assessed by the 10-item Center for Epidemiologic Studies Depression Scale).^37^ Participants were considered to have a history of chronic depression if they reported any indicator of depression on both of the 2 most recent biennial questionnaires.^38^

#### Pandemic-Related Exposures

Secondary exposures included SARS-CoV-2 infection (confirmed by polymerase chain reaction, antigen, or antibody tests), PCC (defined as having >8 weeks of symptoms after initial infection, including fatigue; shortness of breath or difficulty breathing; persistent cough; muscle, joint, or chest pain; smell or taste problems; confusion, disorientation, or brain fog; memory issues; depression, anxiety, or changes in mood; headache; intermittent fever; heart palpitations; rash, blisters, or welts; mouth or tongue ulcers; and other symptoms),^34^ and death of loved ones during the pandemic (yes or no). These exposures were prospectively collected on each of the monthly or quarterly COVID-19 substudy follow-up surveys.

### Outcome Assessment

Cognitive function was assessed with the Cogstate Brief Battery, a computer-administered cognitive test consisting of 4 tasks: detection task (measuring psychomotor function and information processing speed), identification task (measuring visual attention and vigilance), one-back task (measuring working memory), and one card learning task (measuring visual learning and short-term memory).^33,39^ Results for each task were log transformed or arcsine transformed to improve normality.^40^ For each task, scores were standardized (ie, *z* scored) using means and SDs at the wave 1 baseline, with a higher score indicating better cognitive function. We calculated 3 composites from the 4 individual *z*-scored tasks: (1) psychomotor speed and attention, consisting of the mean of the detection and identification tasks; (2) learning and working memory, consisting of the mean of the one card learning and one-back tasks; and (3) a global score, consisting of the mean of all 4 tasks.^40^ Cogstate composite scores have shown high test-retest reliability and correlate well with a clinical diagnosis of Alzheimer disease and mild cognitive impairment.^41^

### Covariates

Covariates were selected a priori based on established association with SARS-CoV-2 infection, PCC, or cognitive function. On the initial cohort questionnaire, participants self-reported birth date, height, and racial identity (Which categories best describe your race? Response options provided by investigators included American Indian or Alaska Native, Asian, Black or African American, Native Hawaiian or Other Pacific Islander, White, or other [including only participants who selected other as their racial identity]). We included race in the analysis because of its known associations with pandemic-related exposures and cognitive outcomes.^42,43,44^ The highest educational level of participants and their parents were collected in 2018 and 2005, respectively. Weight and smoking history were self-reported biennially, and body mass index (BMI) was calculated as weight in kilograms divided by height in meters squared.^2^ Frontline health care worker status (defined as physically working at a site providing health care) was self-reported at the COVID-19 substudy baseline.

### Statistical Analysis

We compared sociodemographic characteristics and cognitive scores of participants with vs without both prepandemic and during-pandemic cognitive assessments. We also compared participant characteristics by wave of enrollment, total number of cognitive assessments, and participation in the COVID-19 substudy (eMethods in Supplement 1). Percentages of missing covariate data were 3% for BMI, 6% for parental educational level, and 19% for participant educational level. A missing indicator was used for categorical covariates; continuous covariates were imputed with the median value in the analytical sample.

Cognitive scores typically improve with practice^45,46,47^; therefore, we considered several methods to account for practice effects (eMethods in Supplement 1).^47^ The best-fitting model included an interaction between the number of prior cognitive assessments (0-7) and time since the previous assessment (6-9, 10-18, or >18 months). More previous cognitive assessments and a shorter interval between consecutive assessments were associated with greater improvement in all 4 tasks, particularly the one card learning task (eTable 3 and eFigure 3 in Supplement 1).

To examine the association of the pandemic with cognitive function within individuals, we fit linear mixed-effects models (with an unstructured correlation matrix) with a random intercept for each participant. The pandemic was the independent variable and was coded as 1 if the cognitive assessment was conducted on or after March 1, 2020, or 0 if it was conducted before March 1, 2020, with each composite score being the dependent variable in separate models. The base model adjusted for age at cognitive assessment (years, linear and squared), number of previous cognitive assessments × time since most recent assessment, wave, and Cogstate platform version (eMethods in Supplement 1). A second model was further adjusted for racial identity (White vs other race [categories combined because of small sample sizes for racial minority groups]), parental and participant educational level, and time-varying BMI and history of disease (diabetes, hypertension, stroke, depression, and cancer, each of which was coded separately). To capture changes in cognitive function more proximate to the pandemic, we performed a sensitivity analysis restricted to wave 2 cognitive assessments (1-3 assessments per person). These assessments were administered in the 2 years before and 2 years after the onset of the pandemic (October 1, 2018, to September 30, 2022).

To evaluate whether the association between the pandemic and cognitive function was modified by risk factors (eg, hypertension and frontline health care worker status), we included a pandemic × risk-factor interaction term in the model, with mutual adjustment for the other risk factors. Statistical significance for additive interaction was estimated using the Wald test. A small number of people in the sample had a stroke (53 of 5191 [1.0%]); therefore, we examined cognitive function in association with only hypertension, diabetes, depression, cancer, and frontline health care worker status. Additionally, we estimated the association of pandemic-related exposures (namely, SARS-CoV-2 infection, PCC, and death of loved ones) with cognitive function by fitting each of the exposures as the independent variable in separate models among participants in the COVID-19 substudy (eMethods in Supplement 1).

We conducted several additional sensitivity analyses. First, we compared population-averaged cognitive function before and during the pandemic among all participants with cognitive assessments (1-8 assessments per person). We used linear regression models with generalized estimating equations (unstructured correlation matrix) with a stabilized inverse probability weight to account for differential loss to follow-up (eMethods in Supplement 1).^48^ Second, we performed multiple imputations with fully conditional specification using 50 imputed datasets to impute covariates.^49^ Third, we adjusted for additional potential confounders: socioeconomic indicators (Census tract percentage with bachelor’s degree and median household income) and lifestyle factors (smoking history, alcohol intake, and physical activity) assessed before the pandemic.

Statistical analyses were performed from January 2023 to January 2025 using SAS, version 9.4 (SAS Institute Inc). All statistical tests were 2-sided, and *P* < .05 was considered statistically significant.

## Results

In total, 20 659 women completed 53 532 Cogstate Brief Battery assessments (median [IQR] 2.0 [1.0-3.0] assessments per person), with 9249 assessments conducted after March 1, 2020. Participants had a mean (SD) age of 62.8 (4.9) years at the first cognitive assessment and 66.4 (4.6) years at the start of the COVID-19 pandemic; 102 identified as American Indian or Alaska Native (0.5%), 223 as Asian (1.1%), 132 as Black or African American (0.6%), 19 as Native Hawaiian or Other Pacific Islander (0.1%), and 20 183 as White (97.7%) (Table 1). Wave 2 new enrollees were, in general, 4 years older than wave 1 enrollees at first assessment, had poorer cognitive function, and had fewer assessments (eTable 4 in Supplement 1). Women who completed both prepandemic and during-pandemic measures (n = 5191; mean [SD] age at first cognitive assessment, 63.0 [4.8] years) contributed 23 678 cognitive assessments (2-8 assessments per person) (Figure 1). Compared with those without both assessments (n = 15 468), these women had a higher educational level, fewer comorbidities, higher learning and working memory scores at first assessment, and 3 more assessments in general (Table 1). Participants with more assessments were younger at first assessment, less likely to have comorbidities, and had better cognitive function (eTable 5 in Supplement 1). Of these 5191 participants, 4456 (85.8%) responded to the COVID-19 substudy, providing 20 415 cognitive assessments (Figure 1).

**Table 1.** Baseline Characteristics of Participants With vs Without Cogstate Cognitive Assessments Both Before and During the COVID-19 Pandemic

Characteristic	Participants, by prepandemic and during-pandemic Cogstate cognitive assessments, No. (%)	
	Without (n = 15 468)^a^	With (n = 5191)
Age, mean (SD), y	62.7 (4.9)	63.0 (4.8)
Racial identity, self-reported		
American Indian or Alaska Native	87 (0.6)	15 (0.3)
Asian	165 (1.1)	58 (1.1)
Black or African American	113 (0.7)	19 (0.4)
Native Hawaiian or Other Pacific Islander	16 (0.1)	3 (0.1)
White	15 087 (97.5)	5096 (98.2)
Parental educational level^b^		
≤High school diploma	7114 (46.0)	2399 (46.2)
Some college	3670 (23.7)	1159 (22.3)
≥Bachelor’s degree	3709 (24.0)	1349 (26.0)
Participant educational level^b^		
Associate’s degree	2819 (18.2)	1082 (20.8)
Bachelor’s degree	4778 (30.9)	2105 (40.6)
≥Graduate school	4162 (26.9)	1839 (35.4)
BMI, mean (SD)	27.6 (6.3)	27.2 (6.2)
Chronic diseases		
Hypertension	6359 (41.1)	2004 (38.6)
Diabetes	1380 (8.9)	401 (7.7)
Cancer	3205 (20.7)	998 (19.2)
Stroke	229 (1.5)	53 (1.0)
Asthma	3220 (20.8)	1056 (20.3)
Depression	3093 (20.0)	1006 (19.4)
*z* Score for psychomotor speed and attention, mean (SD)^c^	−0.05 (0.9)	−0.04 (0.9)
*z* Score for learning and working memory, mean (SD)^c^	−0.07 (0.7)	0.04 (0.7)
No. of tests taken, mean (SD)	1.9 (1.2)	4.6 (1.8)

Abbreviation: BMI, body mass index (calculated as weight in kilograms divided by height in meters squared).

^a^ Overall, 15 090 participants had only prepandemic tests; 378 participants had only during-pandemic tests.

^b^ Percentages may not add up to 100% due to missing data.

^c^ Standardized to wave 1 tests at first cognitive assessment.

Cognitive scores decreased 0.1 to 0.2 SD across 8 years of follow-up except for the one card learning scores, which increased by approximately 0.8 SD, indicating practice effects (eFigure 4 in Supplement 1). After adjusting for practice effects, each 5 years of aging was associated with 0.2 SD lower cognitive scores (eFigure 3 in Supplement 1).

**Figure 2.**
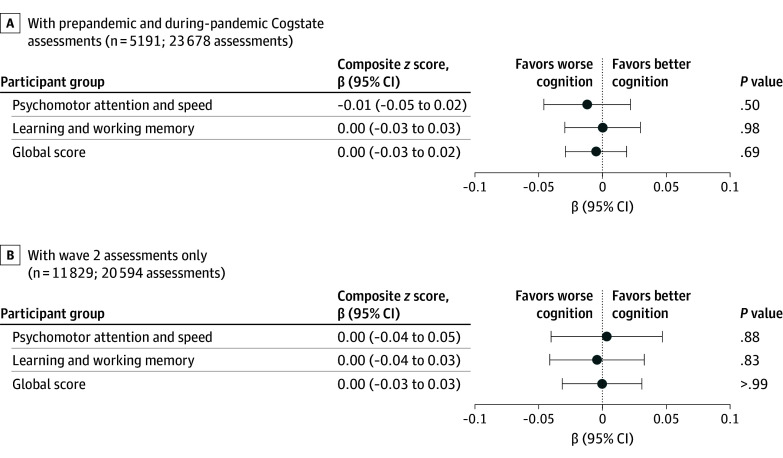
Cogstate Cognitive Composite Scores During vs Before the COVID-19 Pandemic The association of the pandemic with cognitive function was examined using linear mixed-effects models with normal distribution and identity link, unstructured covariance structure, and random intercept for each participant (number of assessments per person: 2-8 for prepandemic and during-pandemic assessments; 1-3 for wave 2 assessments). Pandemic as the independent variable was coded 1 if assessment was on or after or 0 if assessment was before March 1, 2020. The model was adjusted for various factors, including age at baseline; age-squared; time since first test; practice effects (number of tests taken × time since last test); wave; test platform; racial identity; parental educational level; participant educational level; time-varying body mass index; smoking status; and history of diabetes, hypertension, stroke, depression, and cancer. Wave 2 assessments were administered 2 years before and 2 years after the pandemic.

We did not observe a change in cognitive function comparing within-individual prepandemic vs during-pandemic assessments after adjusting for age at cognitive assessment, educational level for both participants and their parents, cognitive test practice effects, comorbidities, wave, and test platform (psychomotor speed and attention: β = −0.01 SD [95% CI, −0.05 to 0.02 SD]; learning and working memory: β = 0.00 SD [95% CI, −0.03 to 0.03 SD]; global score: β = 0.00 SD [95% CI, −0.03 to 0.02 SD]) (eTable 6 in Supplement 1). Results were similar in models further adjusting for racial identity, parental and participant educational level, BMI, and risk factors for cognitive decline (Figure 2). Results were also comparable in analyses restricted to wave 2 assessments (Figure 2; eTable 6 in Supplement 1).

As expected, individuals with cognitive risk factors (hypertension, diabetes, and depression) compared with those without had lower cognitive function both before and during the pandemic (eg, with diabetes vs without diabetes, before the pandemic: β = −0.09 SD [95% CI, −0.15 to −0.03 SD]; during the pandemic: β = −0.08 SD [95% CI, −0.17 to 0.00 SD]). However, we did not observe an interaction (all *P* for interaction >.10) of these risk factors with the pandemic (Table 2). Cognitive function of frontline health care workers was also not differentially affected by the pandemic (1169 of 4456 study participants [26.2%]) vs nonfrontline health care workers.

**Table 2.** Cogstate Cognitive Composite Scores Before and During the COVID-19 Pandemic in Participants With vs Without Risk Factors for Cognitive Decline

Variable	Participants, No.	Cognitive assessments, No.	Global *z* score^a^	
			β (95% CI), SD	*P* value
History of hypertension				
Without hypertension, prepandemic	3187	9145	0.00 [Reference]	NA
With hypertension, prepandemic	2080	5834	−0.03 (−0.07 to 0.00)	.05
Without hypertension, during pandemic	3103	5250	−0.01 (−0.03 to 0.02)	.73
With hypertension, during pandemic	2088	3449	−0.06 (−0.11 to −0.01)	.01
Hypertension × pandemic interaction	NA	NA	−0.02 (−0.06 to 0.01)	.22
History of diabetes				
Without diabetes, prepandemic	4790	13 852	0.00 [Reference]	NA
With diabetes, prepandemic	430	1127	−0.09 (−0.15 to −0.03)	.004
Without diabetes, during pandemic	4758	7980	−0.02 (−0.04 to 0.01)	.23
With diabetes, during pandemic	433	719	−0.08 (−0.17 to 0.00)	.06
Diabetes × pandemic interaction	NA	NA	0.02 (−0.04 to 0.08)	.47
History of depression				
Without depression, prepandemic	4248	12 187	0.00 [Reference]	NA
With depression, prepandemic	1061	2792	−0.03 (−0.07 to 0.01)	.13
Without depression, during pandemic	4197	7075	−0.01 (−0.03 to 0.02)	.56
With depression, during pandemic	994	1624	−0.07 (−0.12 to −0.01)	.02
Depression × pandemic interaction	NA	NA	−0.03 (−0.07 to 0.01)	.16
History of cancer				
Without cancer, prepandemic	4193	12 086	0.00 [Reference]	
With cancer, prepandemic	1086	2893	−0.01 (−0.04 to 0.03)	.76
Without cancer, during pandemic	4098	6860	−0.01 (−0.04 to 0.02)	.40
With cancer, during pandemic	1093	1839	−0.03 (−0.09 to 0.03)	.29
Cancer × pandemic interaction	NA	NA	−0.01 (−0.05 to 0.03)	.54
Frontline health care worker^b^				
Nonfrontline health care worker, prepandemic	3287	9583	0.00 [Reference]	NA
Frontline health care worker, prepandemic	1169	3331	−0.02 (−0.07 to 0.02)	.29
Nonfrontline health care worker, during pandemic	3287	5550	0.00 (−0.03 to 0.03)	.94
Frontline health care worker, during pandemic	1169	1951	−0.05 (−0.11 to 0.01)	.10
Frontline health care worker × pandemic interaction	NA	NA	−0.02 (−0.07 to 0.02)	.24

Abbreviation: NA, not applicable.

^a^ Calculated with a multivariable-adjusted linear mixed-effects model with normal distribution and identity link, unstructured covariance structure, and random intercept for each participant (number of assessments per person: 2-8). Models included terms for time-varying risk factors (depression, diabetes, depression, cancer), time since first test, time-varying risk factors (depression, diabetes, depression, cancer) × time since first test, age at baseline, age-squared, practice effects (number of tests taken × time since last test), wave, test platform, racial identity, parental educational level, participant educational level, time-varying body mass index, and history of stroke.

^b^ Among participants of COVID-19 substudy who reported on health care working status during the pandemic. The model additionally included health care worker status × time since first test.

Participants in the COVID-19 substudy were similar to nonparticipants (eTable 7 in Supplement 1). Cognitive assessments after SARS-CoV-2 infection occurred a median (IQR) of 20.0 (18.5-22.1) months after initial infection. Participants with a history of SARS-CoV-2 infection (164 of 4456 [3.7%]) did not have, groupwise, significantly worse cognitive function than those without infection (global score: β = −0.11 SD; 95% CI, −0.25 to 0.03 SD; *P* = .14) (eFigure 5 in Supplement 1). Participants reporting a history of PCC (62 of 4456 [1.4%]) compared with those without PCC did not have, groupwise, significantly worse cognitive function (global score: β = −0.11 SD; 95% CI, −0.37 to 0.14 SD; *P* = .38). Although the wide CIs indicated considerable uncertainty, point estimates for cognitive decline associated with both SARS-CoV-2 infection and PCC (eg, −0.11 SD) were equivalent to approximately 3 years of cognitive aging in this sample. Death of loved ones was not associated with cognition (global score: β = −0.02 SD; 95% CI, −0.05 to 0.00 SD; *P* = .09). Results were comparable in analyses including all women with a cognitive assessment using multiple imputation and further adjusting for socioeconomic and lifestyle factors (eTable 6, eTable 8, and eTable 9 in Supplement 1).

## Discussion

In this large, prospective, nonclinical cohort study of women with both prepandemic and during-pandemic cognitive assessments, we found no evidence of population-wide cognitive decline during the COVID-19 pandemic, a finding that is contrary to our hypothesis and to anecdotal reports. As expected, the cognitive function of individuals with hypertension, diabetes, and depression was lower than the cognitive function of individuals without these risk factors. However, cognitive function in individuals with these risk factors was not differentially affected by the pandemic, and neither was the cognitive function of frontline health care workers.

Pandemic-related events, including SARS-CoV-2 infection, PCC, and death of loved ones, were not, in a groupwise manner, associated with statistically significant lower cognitive function. Although differences did not reach statistical significance and the low infection rate limited our ability to obtain a precise estimate of the associations, the level of cognitive function in participants with SARS-CoV-2 infection or PCC measured approximately 20 months after initial infection was equivalent to 3 years of aging compared with participants without infection or PCC. This substantively meaningful difference merits further investigation.

Studies have examined the association of cognitive impairment with the pandemic and SARS-CoV-2 infection. A large population-based study (>120 000 participants) conducted among older adults in long-term care facilities observed a lower incidence of cognitive impairment in the first year of the pandemic compared with the prepandemic period,^16^ although these findings may have been affected by survival bias. In contrast, another longitudinal study (>3000 participants) with prepandemic and during-pandemic cognitive assessments found that the pandemic was associated with cognitive decline.^10^ Two large prospective studies found a higher incidence of cognitive impairment or neurocognitive disorders in individuals 6 to 12 months after SARS-CoV-2 infection (compared with those who had never been infected); impairment was more pronounced in those with severe acute-phase symptoms.^20,21^ Several studies have also observed brain structure changes believed to be related to cognitive decline among persons with a history of SARS-CoV-2 infection.^9,50,51^ More recently, a large community study (>141 000 participants) conducted entirely after the pandemic found lower cognitive function in participants with a history of SARS-CoV-2 infection or PCC, with larger deficits observed among participants with ongoing PCC symptoms.^25^

In the present study, we observed no association between lower cognitive function and SARS-CoV-2 infection and PCC. There are several points to consider in the interpretation of the results. First, the sample was population based and included predominantly mild COVID-19 cases (<3.0% required hospitalization^38^), unlike studies involving severe cases. Second, the median time elapsed between infection and cognitive assessment was 20 months; therefore, symptoms may have abated before the assessments. Third, the Cogstate Brief Battery comprises objective measures of cognitive function, which may not capture subjective cognitive experiences, such as brain fog.^52^ It is possible that participants did experience such subjective cognitive symptoms, but it is still reassuring that they did not exhibit decrements on objective assessments. Fourth, the analyses focused on groupwise comparisons among individuals with pandemic-related exposures, and we cannot rule out the possibility of within-group heterogeneity in cognitive function.^6,30^

### Strengths and Limitations

Strengths of this study included a population-based longitudinal design, a large sample size, the ability to contrast prepandemic vs during-pandemic cognitive assessments using a well-validated instrument, and a follow-up duration of up to 2.5 years after the start of the pandemic (which enabled us to characterize cognitive function over a long period). We were able to examine subgroups of participants who may have been at greater risk for pandemic-related cognitive changes. A median of 20 months elapsed between infection and cognitive assessments; therefore, we may have captured longer-lasting outcomes of infection than prior studies.^53,54^

This study has several limitations. First, the data indicate both practice and dropout effects, which may have led to bias. However, results were robust to a variety of statistical adjustments. Second, previous studies have suggested that SARS-CoV-2 infection, PCC, and bereavement affect cognitive function in a dose-dependent manner according to severity or duration^3,20,25^; however, we lacked the data to examine severity or duration of these exposures. Third, due to the limited number of cognitive assessments during follow-up and the lack of a suitable comparison group unexposed to the pandemic, we were unable to use quasiexperimental study designs, such as interrupted time series or difference-in-differences. Fourth, the prepandemic measure of cognitive scores started from 2014, which might not reflect the most recent cognitive function patterns prior to the onset of the pandemic. However, results were similar in analyses using wave 2 cognitive assessments only (October 1, 2018, to September 30, 2022). Fifth, participants were homogenous in race (97.7% identified as White individuals), and all participants were registered nurses at cohort enrollment in 1989, limiting generalizability of the results.

## Conclusions

In this cohort study of middle-aged women, the COVID-19 pandemic and related events were not associated with cognitive function 2.5 years after the onset of the pandemic, even among those with risk factors for cognitive decline. Larger sample sizes and detailed subgroup symptom characterization are needed to better understand the long-term implications of SARS-CoV-2 infection and PCC for cognitive function.

## Notes

Supplement 1.
eMethods
**eFigure 1.** Flowchart of Cognitive Health Sub-Study and COVID-19 Sub-Study, the Nurses’ Health Study II, October 1, 2014 – September 30, 2022 **eFigure 2.** Number of Cogstate Tests Obtained in Each Month of the Follow-up Period **eTable 1.** Characteristics at Enrollment (1989) of Participants Included Versus Excluded in the Cognitive Sub-Study **eTable 2.** Distribution of Total Number of Cognitive Tests During Follow-up **eTable 3.** Estimated Practice Effects by Total Number of Tests Taken and Time Since Last Test **eFigure 3.** Associations Between Age and Cogstate Composite Scores, Without (Panel A-B) and With (Panel C-D) Adjustment for Practice Effects **eTable 4.** Baseline Characteristics (at First Cognitive Assessment) of Eligible Participants by Wave of Enrollment **eTable 5.** Comparing Baseline (First Test) Characteristics of Participants by Total Number of Cognitive Assessments Taken **eFigure 4.** Unadjusted Mean Cognitive Test Scores (Z-Score) by Time Since First Test (Months) **eTable 6.** Association of the COVID-19 Pandemic With Cogstate Composite Scores, Comparing Cognitive Tests Taken During the Pandemic With Those Taken Before the Pandemic **eTable 7.** Comparing Baseline (First Test) Characteristics of Participants Who Did vs Did Not Participate in the COVID-19 Sub-Study, Among the 5,191 Participants With Both Pre- and During- Pandemic Cognitive Assessments **eFigure 5.** Associations of Pandemic-Related Exposures With Cogstate Composite Scores, Among Participants in the COVID-19 Sub-Study and With Both Pre- and During- Pandemic Cognitive Assessments **eTable 8.** Association of the COVID-19 Pandemic With Cogstate Composite Scores, Comparing Cognitive Tests Taken During the Pandemic With Those Taken Before the Pandemic, Using Multiple Imputation **eTable 9.** Associations of Pandemic-Related Exposures With Cogstate Composite Scores, Among Participants in the COVID-19 Sub-Study, With Additional Adjustment for Socioeconomic and Lifestyle Factors

Supplement 2.
Data Sharing Statement
