# Supplementary material for: COVID-19 Pandemic–Related Exposures and Cognitive Function in Middle-Aged Women
Source: JAMA Netw Open. 2025 Apr 17;8(4):e255532. doi: 10.1001/jamanetworkopen.2025.5532 (PMC12006873; doi:10.1001/jamanetworkopen.2025.5532)
Supplement: Supplement 1. — eMethods eFigure 1. Flowchart of Cognitive Health Sub-Study and COVID-19 Sub-Study, the Nurses’ Health Study II, October 1, 2014 – September 30, 2022 eFigure 2. Number of Cogstate Tests Obtained in Each Month of the Follow-up Period eTable 1. Characteristics at Enrollment (1989) of Participants Included Versus Excluded in the Cognitive Sub-Study eTable 2. Distribution of Total Number of Cognitive Tests During Follow-up eTable 3. Estimated Practice Effects by Total Number of Tests Taken and Time Since Last Test eFigure 3. Associations Between Age and Cogstate Composite Scores, Without (Panel A-B) and With (Panel C-D) Adjustment for Practice Effects eTable 4. Baseline Characteristics (at First Cognitive Assessment) of Eligible Participants by Wave of Enrollment eTable 5. Comparing Baseline (First Test) Characteristics of Participants by Total Number of Cognitive Assessments Taken eFigure 4. Unadjusted Mean Cognitive Test Scores (Z-Score) by Time Since First Test (Months) eTable 6. Association of the COVID-19 Pandemic With Cogstate Composite Scores, Comparing Cognitive Tests Taken During the Pandemic With Those Taken Before the Pandemic eTable 7. Comparing Baseline (First Test) Characteristics of Participants Who Did vs Did Not Participate in the COVID-19 Sub-Study, Among the 5,191 Participants With Both Pre- and During- Pandemic Cognitive Assessments eFigure 5. Associations of Pandemic-Related Exposures With Cogstate Composite Scores, Among Participants in the COVID-19 Sub-Study and With Both Pre- and During- Pandemic Cognitive Assessments eTable 8. Association of the COVID-19 Pandemic With Cogstate Composite Scores, Comparing Cognitive Tests Taken During the Pandemic With Those Taken Before the Pandemic, Using Multiple Imputation eTable 9. Associations of Pandemic-Related Exposures With Cogstate Composite Scores, Among Participants in the COVID-19 Sub-Study, With Additional Adjustment for Socioeconomic and Lifestyle Factors [file jamanetwopen-e255532-s001.pdf]

Supplemental Online Content

Wang S, Menor A, Chibnik LB, et al. COVID-19 pandemic–related exposures and cognitive function in middle-aged women. *JAMA Netw Open*. Published online April 17, 2025. doi:10.1001/jamanetworkopen.2025.5532

eMethods

eFigure 1. Flowchart of Cognitive Health Sub-Study and COVID-19 Sub-Study, the Nurses' Health Study II, October 1, 2014 – September 30, 2022

eFigure 2. Number of Cogstate Tests Obtained in Each Month of the Follow-up Period

eTable 1. Characteristics at Enrollment (1989) of Participants Included Versus Excluded in the Cognitive Sub-Study

eTable 2. Distribution of Total Number of Cognitive Tests During Follow-up

eTable 3. Estimated Practice Effects by Total Number of Tests Taken and Time Since Last Test

eFigure 3. Associations Between Age and Cogstate Composite Scores, Without (Panel A-B) and With (Panel C-D) Adjustment for Practice Effects

eTable 4. Baseline Characteristics (at First Cognitive Assessment) of Eligible Participants by Wave of Enrollment

eTable 5. Comparing Baseline (First Test) Characteristics of Participants by Total Number of Cognitive Assessments Taken

eFigure 4. Unadjusted Mean Cognitive Test Scores (Z-Score) by Time Since First Test (Months)

eTable 6. Association of the COVID-19 Pandemic With Cogstate Composite Scores, Comparing Cognitive Tests Taken During the Pandemic With Those Taken Before the Pandemic

eTable 7. Comparing Baseline (First Test) Characteristics of Participants Who Did vs Did Not Participate in the COVID-19 Sub-Study, Among the 5,191 Participants With Both Pre- and During- Pandemic Cognitive Assessments

© 2025 Wang S et al. *JAMA Network Open*

eFigure 5. Associations of Pandemic-Related Exposures With Cogstate Composite Scores, Among Participants in the COVID-19 Sub-Study and With Both Pre- and During-Pandemic Cognitive Assessments

eTable 8. Association of the COVID-19 Pandemic With Cogstate Composite Scores, Comparing Cognitive Tests Taken During the Pandemic With Those Taken Before the Pandemic, Using Multiple Imputation

eTable 9. Associations of Pandemic-Related Exposures With Cogstate Composite Scores, Among Participants in the COVID-19 Sub-Study, With Additional Adjustment for Socioeconomic and Lifestyle Factors

This supplemental material has been provided by the authors to give readers additional information about their work.

eMethods.

Cognitive sub-study study design

In 2014, 43,957 participants who had responded to a 2008 supplemental questionnaire were invited to take part in a cognitive health sub-study, with assessments at 6- or 12-month intervals, for up to 24 months (15,138 [34%] enrolled, 'wave 1'). In 2018 ('wave 2'), another wave of sub-study invitations was sent to 37,682 participants who completed a 2018 supplemental questionnaire. Of these, 11,920 (32%) enrolled. Wave 2 cognitive assessments were conducted every 12 months, for up to 24 months.

Comparison of participants characteristics

We also compared sociodemographic characteristics and cognitive scores of participants in wave 1 with those of the new participants in wave 2 at their first cognitive assessment and among women who completed different numbers of cognitive assessments during follow-up (range, 1–8). Lastly, we compared characteristics among women who did versus did not participate in the COVID-19 sub-study.

Practice effects of cognitive testing

We considered several methods of accounting for practice effects, including using an indicator for the first assessment (coded: 0, 1), adjusting for number of prior assessments (0–7), adjusting for the square root of number of prior assessments; with each of these methods we also included elapsed time since the previous assessment (coded: 6–9, 10–18, >18 months).

Conservative ascertainment of SARS-CoV-2 infection and long COVID

To estimate the SARS-CoV-2 infection and long COVID exposures conservatively, we: 1) included only COVID-19 cases that had been confirmed with tests; and 2) further excluded cognitive assessments conducted after the return of the final COVID-19 sub-study questionnaire among women who never reported SARS-CoV-2 infection, because their infection status following this questionnaire was unknown.

Changes in Cogstate test software

In April 2022, Cogstate software was updated from version 7 to version 8. There has been no change to the test contents. The primary update in version 8 is that the test that can now be administered on a tablet (previously it was only available on a computer). This update also eliminated the need for Adobe Flash to complete the tests, reducing the technical barriers for conducting assessments. A small proportion of the tests were conducted on the new version 8 (4,100 out of 53,532 tests that passed quality check, 7.6%). We included the Cogstate version (8 versus 7) variable in all models.

Inverse probability weighting for selective attrition

To account for differential loss to follow-up by cognitive function, we created a stabilized inverse probability weight for selective attrition. Briefly, weights were constructed using variables associated with loss to follow-up, including participant and parents' educational attainment, time-updated age (years, linear and squared), wave, BMI, hypertension, asthma, and cognitive scores in the previous assessment. Weights were calculated as the inverse of the conditional probability of remaining in the study.

eFigure 1. Flowchart of cognitive health sub-study and COVID-19 sub-study, the Nurses' Health Study II, October 1, 2014 – September 30, 2022.

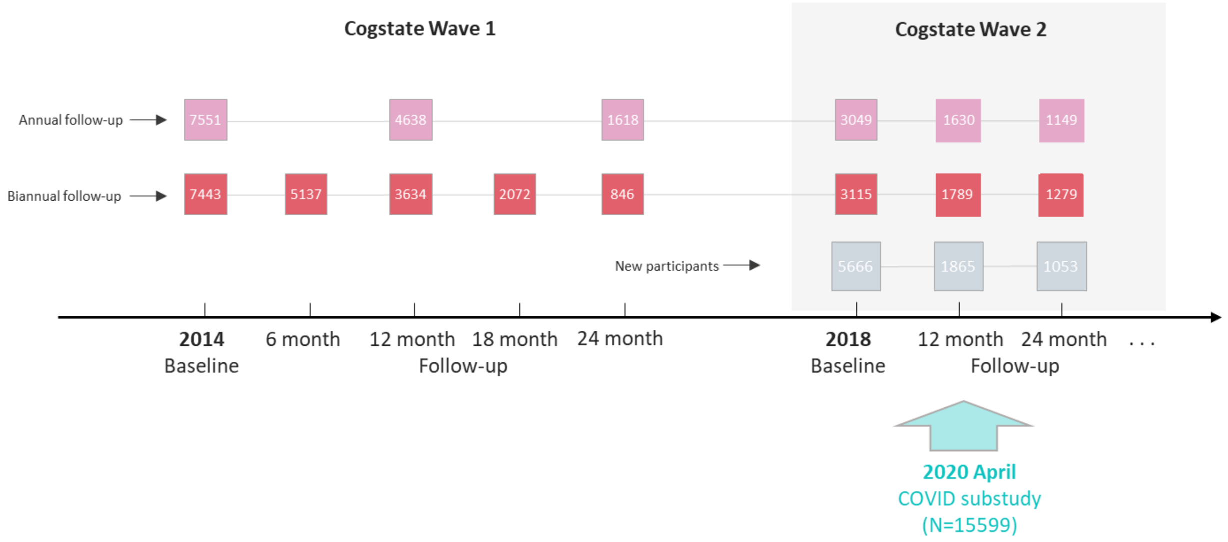

Note. All groups are non-overlapping. Participants of Wave 1 were randomized at baseline to receive either annual or biannual follow-up.

© 2025 Wang S et al. *JAMA Network Open*

eFigure 2. Number of Cogstate tests obtained in each month of the follow-up period.

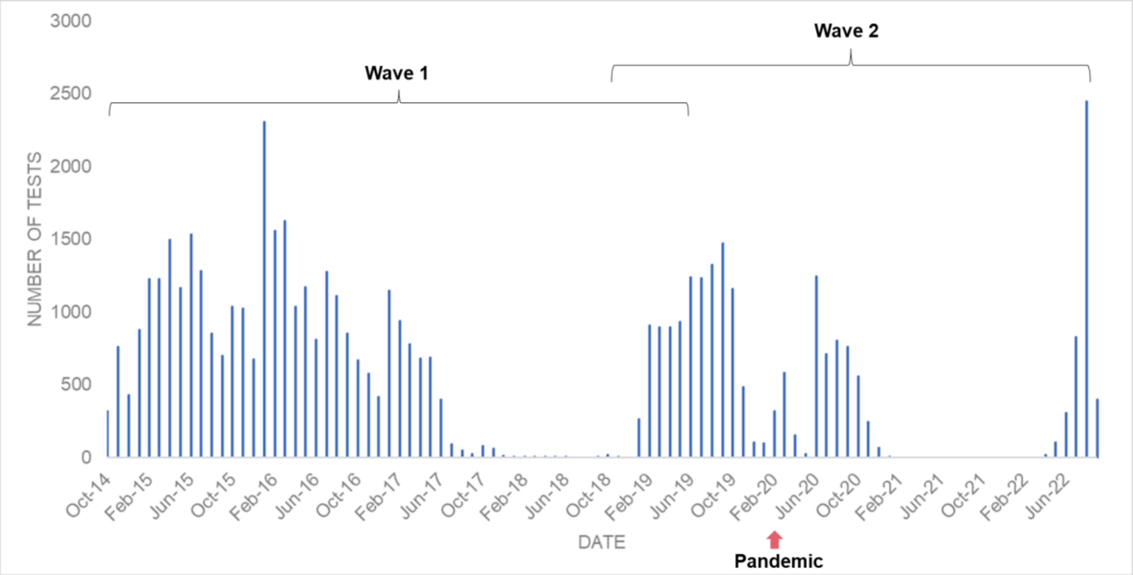

Note. Blue arrow indicates the start of the COVID-19 pandemic (March 1, 2020). Wave 1 tests were conducted between October 1, 2014, and July 31, 2019. Wave 2 tests were conducted between October 1, 2018, and September 30, 2022. In total, 20,659 participants (6,164 in both waves) completed 53,532 tests; 9,249 tests (17%) were conducted after March 1, 2020.

© 2025 Wang S et al. *JAMA Network Open*

eTable 1. Characteristics at enrollment (1989) of participants included versus excluded in the cognitive sub-study.

Characteristics	No. (%)		
	Not included in the cognitive sub-study n=95,770	Included in the cognitive sub-study n=20,659	
		Have both pre- and during- pandemic Cogstate measurements	
		No (n=15,468)	Yes (n=5,191)
Age, mean (SD), y	34.3 (4.7)	34.8 (4.6)	34.9 (4.5)
Racial identity			
American Indian/Alaska Native	358 (0.4)	87 (0.6)	15 (0.3)
Asian	2,092 (2.2)	165 (1.1)	58 (1.1)
Black/African American	2,176 (2.3)	113 (0.7)	19 (0.4)
Native Hawaiian/Pacific Islander	97 (0.1)	16 (0.1)	3 (0.1)
White	91,026 (95.1)	15,807 (97.5)	5,096 (98.2)
Marital status	73,885 (77.2)	12,103 (78.3)	3,980 (76.7)
Census tract median household income, mean (SD), USD	59,682.6 (22,237.4)	61,645.3 (22,595.6)	62,536.4 (22,237.4)
Census tract % population with bachelor's degree or higher, mean (SD)	28.8 (17.1)	30.9 (17.6)	31.8 (17.5)
BMI, mean (SD), kg/m^2	24.2 (5.1)	23.9 (4.8)	23.6 (4.6)
Smoking status			
Never smoker	61,904 (64.8)	10,281 (66.5)	3,456 (66.6)
Past smoker	20,238 (21.2)	3,457 (22.4)	1,176 (22.7)
Current smoker	13,464 (14.1)	1,714 (11.1)	556 (10.7)
Disease history			
Hypertension, yes	5,534 (5.8)	744 (4.8)	232 (4.5)
Diabetes, yes	880 (0.9)	74 (0.5)	18 (0.4)
Stroke, yes	285 (0.3)	25 (0.2)	11 (0.2)
Cancer, yes	867 (0.9)	134 (0.9)	41 (0.8)

© 2025 Wang S et al. *JAMA Network Open*

eTable 2. Distribution of total number of cognitive tests during follow-up.

Number of tests	No. (%) persons
**Total number of tests**	
1	7,755 (37.5)
2	4,262 (20.6)
3	3,602 (17.4)
4	1,733 (8.4)
5	1,445 (7.0)
6	1,072 (5.2)
7	462 (2.2)
8	328 (1.6)
**Number of tests before the COVID-19 pandemic**	
0	378 (1.8)
1	9,206 (44.6)
2	4,069 (19.7)
3	3,283 (15.9)
4	2,130 (10.3)
5	1,024 (5.0)
6	533 (2.6)
7	36 (0.2)
**Number of tests during the COVID-19 pandemic**	
0	15,090 (73.0)
1	1,889 (9.1)
2	3,680 (17.8)

© 2025 Wang S et al. *JAMA Network Open*

eTable 3. Estimated practice effects by total number of tests taken and time since last test.

	z-score of psychomotor attention and speed		z-score of learning and working memory	
No. test (both waves)	z-score of detection task	z-score of identification task	z-score of one card back task	z-score of one card learning task
	β (95% CI), SD[a]			
1st test	Ref	Ref	Ref	Ref
2nd test, 6-9 months	0.29 (0.27-0.32)	0.14 (0.11-0.16)	0.12 (0.09-0.14)	0.39 (0.36-0.42)
2nd test, 10-18 months	0.27 (0.25-0.30)	0.14 (0.12-0.16)	0.10 (0.08-0.12)	0.40 (0.37-0.42)
2nd test, >18 months	0.21 (0.15-0.27)	0.12 (0.07-0.18)	0.17 (0.11-0.22)	0.25 (0.18-0.31)
3rd test, 6-9 months	0.33 (0.31-0.36)	0.17 (0.14-0.19)	0.21 (0.19-0.24)	0.62 (0.59-0.66)
3rd test, 10-18 months	0.30 (0.27-0.34)	0.14 (0.11-0.18)	0.17 (0.14-0.20)	0.54 (0.50-0.58)
3rd test, >18 months	0.21 (0.15-0.27)	0.13 (0.08-0.18)	0.22 (0.17-0.27)	0.38 (0.33-0.44)
4th test, 6-9 months	0.41 (0.37 0.44)	0.21 (0.18-0.25)	0.34 (0.31-0.38)	0.82 (0.78-0.86)
4th test, 10-18 months	0.40 (0.32-0.47)	0.20 (0.14-0.27)	0.29 (0.23-0.36)	0.76 (0.69-0.83)
4th test, >18 months	0.26 (0.19-0.32)	0.15 (0.09-0.21)	0.29 (0.23-0.35)	0.53 (0.46-0.60)
5th test, 6-9 months	0.39 (0.34-0.44)	0.18 (0.13-0.22)	0.32 (0.28-0.36)	0.92 (0.86-0.98)
5th test, 10-18 months	0.46 (0.39-0.53)	0.22 (0.15-0.28)	0.30 (0.24-0.36)	0.86 (0.78-0.93)
5th test, >18 months	0.33 (0.25-0.40)	0.21 (0.14-0.28)	0.34 (0.27-0.41)	0.60 (0.51-0.68)
6th test, 6-9 months	N/A	N/A	N/A	N/A
6th test, 10-18 months	0.50 (0.41-0.60)	0.28 (0.20-0.36)	0.36 (0.28-0.44)	0.95 (0.84-1.05)
6th test, >18 months	0.37 (0.29-0.45)	0.23 (0.15-0.31)	0.41 (0.33-0.48)	0.72 (0.63-0.82)
7th test, 6-9 months	N/A	N/A	N/A	N/A
7th test, 10-18 months	0.60 (0.50-0.69)	0.28 (0.19-0.37)	0.50 (0.42-0.59)	1.10 (0.99-1.20)
7th test, >18 months	0.46 (0.34-0.59)	0.27 (0.16-0.38)	0.46 (0.35-0.57)	0.82 (0.69-0.95)
8th test, 6-9 months	N/A	N/A	N/A	N/A
8th test, 10-18 months	N/A	N/A	N/A	N/A
8th test, >18 months	0.52 (0.40-0.65)	0.27 (0.15-0.38)	0.57 (0.47-0.68)	0.89 (0.76-1.03)

Abbreviations: zPsyAtt, z-score of psychomotor attention and speed; zDET, z-score of detection task; zIDN, z-score of identification task; zLearnMem, z-score of learning and working memory; zONB, z-score of one card back task; zOCL, z-score of one card learning task. Inverse probability weighting of attrition applied.

[a] Generalized estimating equation model with normal distribution and identity link, unstructured covariance structure, adjusted for age at assessment, age squared, pandemic (pandemic = 1 if test was taken after March 1, 2020; pandemic = 0 if test was taken before March 1, 2020), platform version, and wave. The interpretation of the β coefficient is the difference in Cogstate z-score compared to the first test, for which higher values suggesting greater practice effects.

eFigure 3. Associations between age and Cogstate composite scores, without (Panel A-B) and with (Panel C-D) adjustment for practice effects.

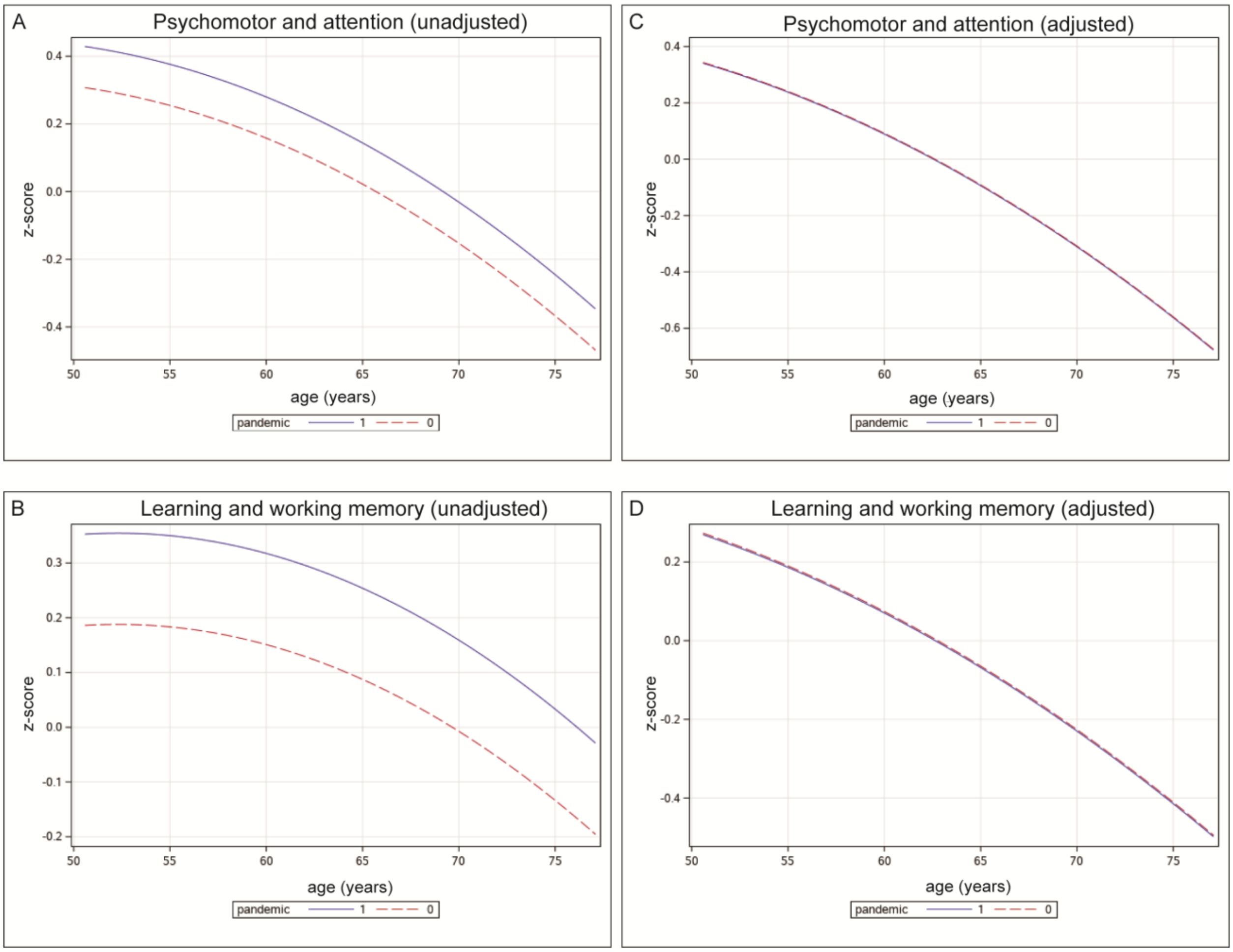

Abbreviations: zPsyAtt, z-score of psychomotor attention and speed; zLearnMem, z-score of learning and working memory

Generalized estimating equation model with normal distribution and identity link, unstructured covariance structure, adjusted for pandemic (pandemic = 1 if test was taken after March 1, 2020; pandemic = 0 if test was taken before March 1, 2020), platform version, and wave. Inverse probability weighting of attrition applied. Fitted trajectories were calculated for cognitive assessments taken in version 7 and wave 1.

Practice effects modelled as number of tests taken*time-since-last-test.

© 2025 Wang S et al. *JAMA Network Open*

eTable 4. Baseline characteristics (at first cognitive assessment) of eligible participants by wave of enrollment.

Characteristics	No. (%)	
	Wave 1 (Began in 2014) n=14,993	New participants in Wave 2 (Began in 2018) n=5,666
Age, mean (SD), y	61.7 (4.6)	65.7 (4.6)
Racial identity, White[a]	14,648 (97.7)	5,535 (97.7)
Parents' highest education[b]		
High school or lower	7,022 (46.8)	2,491 (44.0)
Some college	3,592 (24.0)	1,237 (21.8)
College or graduate school	3,680 (24.5)	1,378 (24.3)
Participant's education attainment[b]		
Associate's degree	2,588 (17.3)	1,313 (23.2)
College	4,638 (30.9)	2,245 (39.6)
Graduate school	4,100 (27.4)	1,901 (33.6)
BMI, mean (SD), kg/m^2	27.5 (6.3)	27.6 (6.3)
Disease history		
Hypertension, yes	5,974 (39.9)	2,389 (42.2)
Diabetes, yes	1,199 (8.0)	582 (10.3)
Stroke, yes	213 (1.4)	69 (1.2)
Depression, yes	2,950 (19.7)	1,149 (20.3)
Cancer, yes	2,996 (20.0)	1,207 (21.3)
Z-score of psychomotor speed and attention, mean (SD)[c]	0.00 (0.9)	-0.18 (1.0)
Z-score of learning and working memory, mean (SD)[c]	0.00 (0.7)	-0.16 (0.7)
Number of tests taken during follow-up, mean (SD)	3.0 (1.9)	1.5 (0.8)

[a] Only White category was presented because other racial categories had very small numbers.
[b] Percentages may not add up to 100% due to missingness.
[c] Standardized to wave 1 tests at first assessment.

© 2025 Wang S et al. *JAMA Network Open*

eTable 5. Comparing baseline (first test) characteristics of participants by total number of cognitive assessments taken.

Characteristics	No. (%)				
	Total tests=1 n=7,755	Total tests=2 n=4,262	Total tests=3 n=3,602	Total tests=4 n=1,733	Total test≥5 n=3,307
Age, mean (SD), y	63.5 (5.1)	62.5 (4.8)	62.9 (4.9)	61.9 (4.5)	61.9 (4.6)
Race, White[a]	7,553 (97.4)	4,155 (97.5)	3,529 (98.0)	1,697 (97.9)	3,249 (98.3)
Parents' highest education[b]					
High school or less	3,458 (44.6)	1,965 (46.1)	1,693 (47.0)	834 (48.1)	1,563 (47.3)
Some college	1,816 (23.4)	1,017 (23.9)	833 (23.1)	402 (23.2)	761 (23.0)
College or graduate school	1,867 (24.1)	1,033 (24.2)	892 (24.8)	412 (23.8)	854 (25.8)
Participant's education attainment[b]					
Associate's degree	1,395 (18.0)	801 (18.8)	679 (18.9)	316 (18.2)	710 (21.5)
College	2,396 (30.9)	1,290 (30.3)	1,268 (35.2)	636 (36.7)	1,293 (39.1)
Graduate school	2,101 (27.1)	1,167 (27.4)	1,056 (29.3)	553 (31.9)	1,124 (34.0)
BMI, mean (SD), kg/m^2	27.7 (6.3)	27.6 (6.3)	27.4 (6.3)	27.4 (6.3)	27.1 (6.2)
Chronic diseases					
Hypertension, yes	3,283 (42.3)	1,746 (41.0)	1,418 (39.4)	674 (38.9)	1,242 (37.6)
Diabetes, yes	760 (9.8)	358 (8.4)	298 (8.3)	145 (8.4)	220 (6.7)
Cancer, yes	1,609 (20.8)	904 (21.2)	706 (19.6)	352 (20.3)	632 (19.1)
Stroke, yes	112 (1.4)	67 (1.6)	44 (1.2)	24 (1.4)	35 (1.1)
Asthma, yes	1,664 (21.5)	955 (22.4)	674 (18.7)	364 (21.0)	619 (18.7)
Depression, yes	1,562 (20.1)	835 (19.6)	728 (20.2)	338 (19.5)	636 (19.2)
z-score of psychomotor speed and attention, mean (SD)[c]	-0.11 (1.0)	-0.02 (0.9)	-0.03 (0.9)	-0.01 (0.9)	0.01 (0.9)
z-score of learning and working memory, mean (SD)[c]	-0.14 (0.8)	-0.04 (0.7)	-0.01 (0.7)	0.04 (0.7)	0.09 (0.7)

[a] Only White category was presented because other racial categories had very small numbers.

[b] Percentages may not add up to 100% due to missingness.

[c] Standardized to wave 1 tests at enrollment.

© 2025 Wang S et al. *JAMA Network Open*

eFigure 4. Unadjusted mean cognitive test scores (z-score) by time since first test (months).

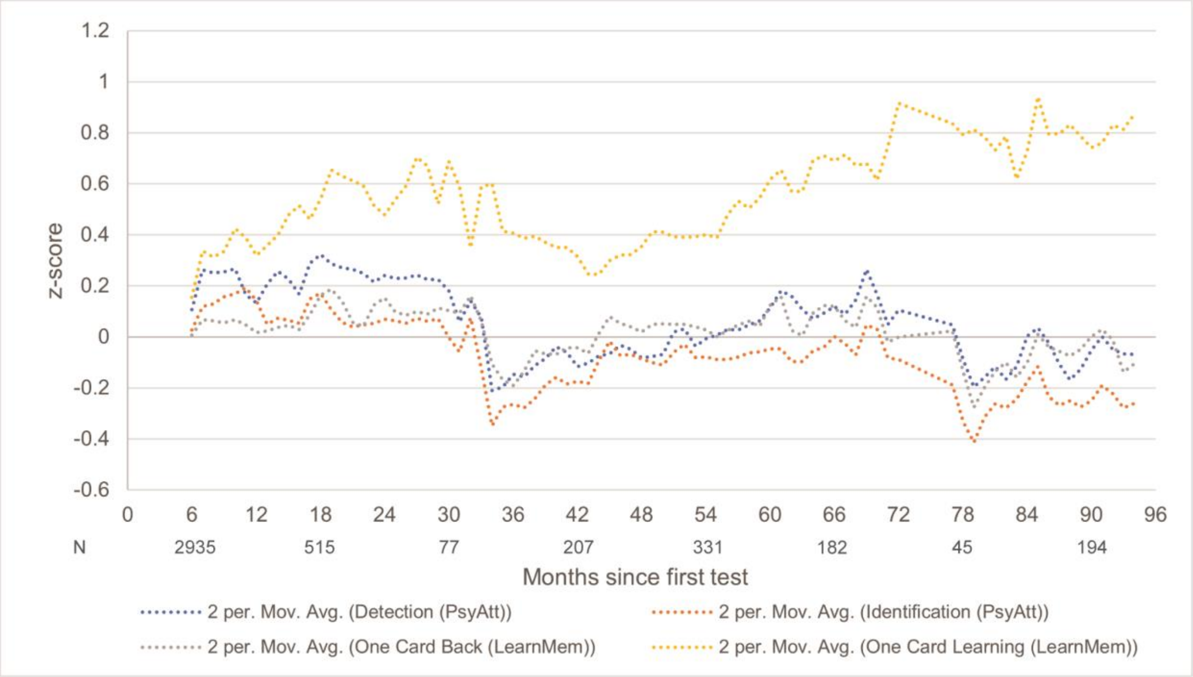

Abbreviations: 2 per. Mov. Ave, 2 periods (months) of moving average; PsyAtt, psychomotor attention and speed; LearnMem, learning and working memory.
Note. There is no average value before 6 months because the second tests were taken starting at 6 months. Months with less than 10 assessments were not included in the plot.

eTable 6. Association of the COVID-19 pandemic with Cogstate composite scores, comparing cognitive tests taken during the pandemic with those taken before the pandemic.

	N persons/ N assessments	Model 1: Adjusted for age at first test, age squared, time since first test, practice effects, wave, and test platform[a]		Model 2: Further adjusted for racial identity, parents' and participant's education, and time-varying health covariates[b]	
		β (95% CI), SD	*P* value	β (95% CI), SD	*P* value
**Restricted to participants with both pre- and during-pandemic cognitive assessments (2014-2022)[c]**	5,191/23,678				
Psychomotor speed and attention z-score		-0.01 (-0.05 to 0.02)	.50	-0.01 (-0.05 to 0.02)	.50
Learning and working memory z-score		0.00 (-0.03 to 0.03)	.86	0.00 (-0.03 to 0.03)	.98
Global cognitive function z-score		0.00 (-0.03 to 0.02)	.76	0.00 (-0.03 to 0.02)	.69
**Wave 2 assessments only (2018-2022)[d]**	11,829/20,594				
Psychomotor speed and attention z-score		0.01 (-0.04 to 0.05)	.82	0.00 (-0.04 to 0.05)	.88
Learning and working memory z-score		0.00 (-0.04 to 0.04)	.93	0.00 (-0.04 to 0.03)	.83
Global cognitive function z-score		0.00 (-0.03 to 0.03)	.85	0.00 (-0.03 to 0.03)	>.99
**All participants (2014-2022)[e]**	20,659/53,532				
Psychomotor speed and attention z-score		0.00 (-0.03 to 0.03)	.87	0.00 (-0.03 to 0.03)	.88
Learning and working memory z-score		0.00 (-0.03 to 0.02)	.78	-0.01 (-0.03 to 0.02)	.71
Global cognitive function z-score		0.00 (-0.02 to 0.02)	.79	0.00 (-0.02 to 0.02)	.83

[a] Model 1: adjusted for age at baseline, age-squared, time since first test, practice effects (number of tests taken*time since last test), wave, test platform.

[b] Model 2: Model 1 + racial identity, parents' education, participant's education, and time-varying BMI, smoking status, history of diabetes, hypertension, stroke, depression, and cancer

[c] Linear mixed effects model with normal distribution and identity link, unstructured covariance structure, and random intercept for each participant (n assessments per person: 2-8). Pandemic was coded as a variable (pandemic = 1 if test was taken after March 1, 2020; pandemic = 0 if test was taken before March 1, 2020).

[d] Linear mixed effects model with normal distribution and identity link, unstructured covariance structure, and random intercept for each participant (n assessments per person: 1-3). Pandemic was coded as a variable (pandemic = 1 if test was taken after March 1, 2020; pandemic = 0 if test was taken before March 1, 2020),

[e] Generalized estimating equation model with normal distribution and identity link, unstructured covariance structure (n assessments per person: 1-8). Pandemic was coded as a variable (pandemic = 1 if test was taken after March 1, 2020; pandemic = 0 if test was taken before March 1, 2020). Inverse probability weighting of attrition was applied.

© 2025 Wang S et al. *JAMA Network Open*

eTable 7. Comparing baseline (first test) characteristics of participants who did vs did not participate in the COVID-19 sub-study, among the 5,191 participants with both pre- and during- pandemic cognitive assessments.

Characteristics	No. (%)	
	**Not in COVID-19 sub-study n=735**	**In COVID-19 sub-study n=4,456**
Age, mean (SD), y	63.0 (4.9)	63.0 (4.8)
Race, White[a]	723 (98.4)	4,373 (98.1)
Parents' highest education[b]		
High school or lower	351 (47.8)	2,048 (46.0)
Some college	177 (24.1)	982 (22.0)
College or graduate school	165 (22.5)	1,184 (26.6)
Participant's education attainment[b]		
Associate's degree	156 (21.2)	926 (20.8)
College	306 (41.6)	1,799 (40.4)
Graduate school	235 (32.0)	1,604 (36.0)
Wave of first assessment		
Wave 1	482 (65.6)	3,062 (68.7)
Wave 2	253 (34.4)	1,394 (31.3)
BMI, mean (SD), kg/m^2	27.2 (6.3)	27.2 (6.2)
Chronic diseases		
Hypertension, yes	277 (37.7)	1,727 (38.8)
Diabetes, yes	63 (8.6)	338 (7.6)
Cancer, yes	150 (20.4)	848 (19.0)
Stroke, yes	12 (1.6)	41 (0.9)
Asthma, yes	154 (21.0)	902 (20.2)
Depression, yes	146 (19.9)	860 (19.3)
z-score of psychomotor speed and attention, mean (SD)[c]	-0.07 (0.9)	-0.03 (0.7)
z-score of learning and working memory, mean (SD)[c]	0.00 (0.8)	0.04 (0.7)
Number of tests, mean (SD)	4.4 (1.8)	4.6 (1.8)

[a] Only White category was presented because other racial categories had very small numbers.
[b] Percentages may not add up to 100% due to missingness.
[c] Standardized to wave 1 tests at enrollment.

© 2025 Wang S et al. *JAMA Network Open*

eFigure 5. Associations of pandemic-related exposures with Cogstate composite scores, among participants in the COVID-19 sub-study and with both pre- and during- pandemic cognitive assessments.

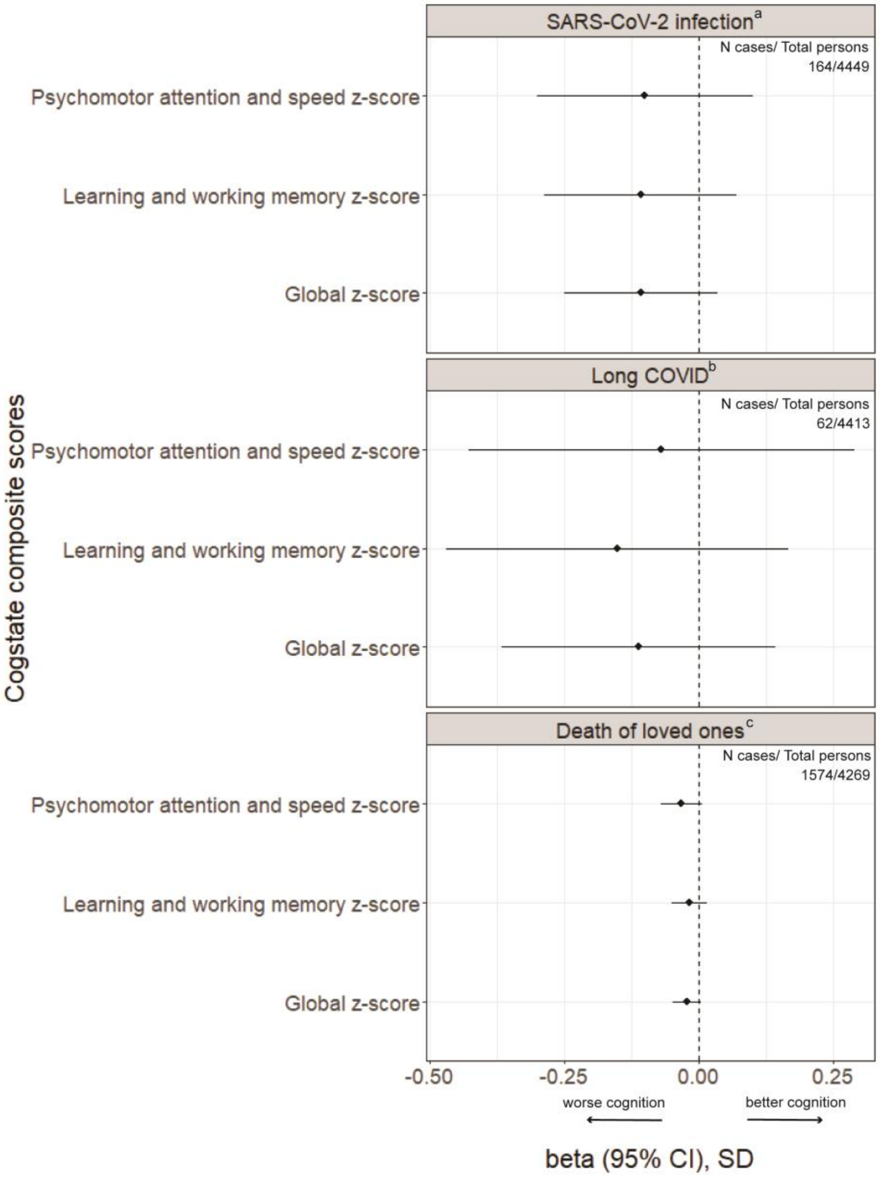

[a] Cogstate tests conducted after the final follow-up survey from participants who never reported having been infected with SARS-CoV-2 during the COVID-19 sub-study were excluded (n=3,295 tests).

[b] Cogstate tests conducted after the final follow-up survey from participants who never reported having been infected with SARS-CoV-2 and/or long COVID during the COVID-19 sub-study were excluded (n=3,515 tests).

[c] Cogstate tests conducted during the COVID-19 sub-study for participants reported ever experiencing death of loved ones, and tests conducted after the final follow-up survey from participants who never reported having experienced death of loved ones were excluded (n=2,265 tests).

Linear mixed effects model with normal distribution and identity link, unstructured covariance structure, and random intercept for each participant (n assessments per person: 2-8). Pandemic was coded as a variable (pandemic = 1 if test was taken after March 1, 2020; pandemic = 0 if test was taken before March 1, 2020). Models included terms for exposure, time-since-first-test, age at baseline, age-squared, practice effects (number of tests taken*time since last test), wave, test platform, racial identity, parents' education, participant's education, and time-varying BMI, history of diabetes, hypertension, cancer, stroke, depression.

© 2025 Wang S et al. *JAMA Network Open*

eTable 8. Association of the COVID-19 pandemic with Cogstate composite scores, comparing cognitive tests taken during the pandemic with those taken before the pandemic, using multiple imputation.

		Main analysis[a]		Using multiple imputation with fully conditional specification[a]	
	N persons/ N assessments	β (95% CI), SD	*P* value	β (95% CI), SD	*P* value
**Restricted to participants with both pre- and during-pandemic cognitive assessments (2014-2022)**	5,191/23,678				
Psychomotor speed and attention z-score		-0.01 (-0.05 to 0.02)	.50	-0.01 (-0.05 to 0.02)	.49
Learning and working memory z-score		0.00 (-0.03 to 0.03)	.98	0.00 (-0.03 to 0.03)	.98
Global cognitive function z-score		0.00 (-0.03 to 0.02)	.69	0.00 (-0.03 to 0.02)	.69

[a] Linear mixed effects model with normal distribution and identity link, unstructured covariance structure, and random intercept for each participant (n assessments per person: 2-8). Pandemic was coded as a variable (pandemic = 1 if test was taken after March 1, 2020; pandemic = 0 if test was taken before March 1, 2020). Models adjusted for age at baseline, age-squared, time since first test, practice effects (number of tests taken*time since last test), wave, test platform, racial identity, parents' education, participant's education, and time-varying BMI, smoking status, history of diabetes, hypertension, stroke, depression, and cancer.

© 2025 Wang S et al. *JAMA Network Open*

eTable 9. Association of the COVID-19 pandemic with Cogstate composite scores, comparing cognitive tests taken during the pandemic with those taken before the pandemic, with additional adjustment for socioeconomic and lifestyle factors.

		**Main analysis[a]**		**Additionally adjusted for neighborhood % with bachelor's degree, neighborhood census household income, smoking history, alcohol intake, and physical activity**	
	**N cases/ N persons**	**β (95% CI), SD**	***P* value**	**β (95% CI), SD**	***P* value**
**SARS-CoV-2 infection[b]**	164/4,449				
Psychomotor speed and attention z-score		-0.10 (-0.30 to 0.10)	.32	-0.10 (-0.30 to 0.10)	.33
Learning and working memory z-score		-0.11 (-0.29 to 0.07)	.23	-0.11 (-0.29 to 0.07)	.23
Global cognitive function z-score		-0.11 (-0.25 to 0.03)	.14	-0.11 (-0.25 to 0.03)	.14
**Long COVID[c]**	62/4,413				
Psychomotor speed and attention z-score		-0.07 (-0.43 to 0.29)	.70	-0.07 (-0.43 to 0.29)	.71
Learning and working memory z-score		-0.15 (-0.47 to 0.17)	.35	-0.15 (-0.47 to 0.16)	.34
Global cognitive function z-score		-0.11 (-0.37 to 0.14)	.38	-0.11 (-0.37 to 0.14)	.38

[a] Models adjusted for time-since-first-test, age at baseline, age-squared, practice effects (number of tests taken*time since last test), wave, test platform, racial identity, parents' education, participant's education, and time-varying BMI, history of diabetes, hypertension, cancer, stroke, depression.

[b] Cogstate tests conducted after the final follow-up survey from participants who never reported having been infected with SARS-CoV-2 during the COVID-19 sub-study were excluded (n=3,295 tests).

[c] Cogstate tests conducted after the final follow-up survey from participants who never reported having been infected with SARS-CoV-2 and/or long COVID during the COVID-19 sub-study were excluded (n=3,515 tests).

© 2025 Wang S et al. *JAMA Network Open*
