# Supplementary material for: COVID-19 Pandemic–Related Exposures and Cognitive Function in Middle-Aged Women
Source: JAMA Netw Open. 2025 Apr 17;8(4):e255532. doi: 10.1001/jamanetworkopen.2025.5532 (PMC12006873; doi:10.1001/jamanetworkopen.2025.5532)
Supplement: Supplement 2. — Data Sharing Statement [file jamanetwopen-e255532-s002.pdf]

Data Sharing Statement

Wang. COVID-19 Pandemic–Related Exposures and Cognitive Function in Middle-Aged Women. *JAMA Netw Open*. Published April 17, 2025.
doi:10.1001/jamanetworkopen.2025.5532

Data
Data available: No

Additional Information
Explanation for why data not available: Further information including the procedures to obtain and access data from the Nurses' Health Studies is described at https://www.nurseshealthstudy.org/researchers (contact email: nhsaccess@channing.harvard.edu).
